# Advances in proton therapy technology and global clinical applications

**DOI:** 10.3389/fonc.2026.1718677

**Published:** 2026-02-11

**Authors:** Qi Zhang, Wencui Yang, Lina Tan, Xiangyu Guo, Tao Wang, Pengfei Zhu, Zhouchen Jing, Lei Ma, Jun Hou

**Affiliations:** Proton Center, Xi’an International Medical Center Hospital, Xi’an, Shaanxi, China

**Keywords:** artificial intelligence, clinical application, FLASH radiotherapy, pencil beam scanning, proton therapy

## Abstract

Proton therapy, by leveraging its unique physical characteristic of the Bragg peak, enables high-precision dose delivery to the tumor target while effectively protecting surrounding normal tissues, and has become an important representative of advanced radiotherapy. This review aims to systematically summarize key technological breakthroughs in recent years that have driven the progress of proton therapy, including compact superconducting accelerators, pencil beam scanning (PBS), image-guided proton therapy (IGPT), and the transformative ultra-high dose rate FLASH radiotherapy, while highlighting the role of artificial intelligence (AI) in advancing proton therapy toward real-time adaptive precision radiotherapy. The article also explores the global distribution and development status of proton centers, with a specific analysis of China’s notable advancements as an emerging market in center construction, equipment localization, and the treatment of characteristic local tumor types. Moving forward, it is essential to continue promoting technological integration and innovation, strengthen high-quality clinical research, and develop a more accessible, intelligent, and personalized proton therapy system to achieve broader clinical application and patient benefit.

## 1 Introduction

The physical characteristic of the Bragg peak in proton therapy enables the precise concentration of radiation dose within the tumor target while maximizing the protection of surrounding healthy tissues (1–3). Unlike photon (X-ray) radiotherapy, which exhibits exponential attenuation in human tissues and delivers unnecessary radiation dose to surrounding tissues, the proton beam loses energy slowly upon entering the body, releasing most of its energy near the end of its range. This creates a sharp dose peak within the target, followed by a rapid dose fall-off to nearly zero (4). This unique depth-dose distribution not only significantly reduces radiation exposure to normal tissues before and beyond the tumor, achieving “high-precision, low-damage” dose delivery, but also provides a physical basis for treating tumors in complex anatomical sites or highly radiation-sensitive pediatric tumors. In recent years, revolutionary breakthroughs and intelligent trends in proton therapy technology have further enhanced the advantages of proton dose distribution conformity and normal tissue protection compared to traditional intensity-modulated radiation therapy (IMRT) (5).

The development of proton therapy represents a history of innovation from physical concept to mainstream clinical application. Since the concept was first proposed by Robert R. Wilson in 1946, and the first human application of proton beams was realized in the 1950s at the Berkeley Radiation Laboratory in the United States, the field has undergone early technical exploration and facility limitations (6). The establishment of the world’s first hospital-based proton therapy center at Loma Linda University Medical Center in 1990 marked the beginning of the clinical era of proton therapy (7). Entering the 21st century, with the maturation of compact accelerator platforms based on superconducting technology, proton therapy equipment has shown significant trends toward miniaturization, superconductivity, and intelligence, gradually moving from research institutions to general hospitals, accompanied by rapid growth in the number of clinical cases. According to the latest statistics from the Particle Therapy Co-operative Group (PTCOG), by the end of 2024, there were over 120 operational proton therapy centers worldwide, primarily distributed across North America, Europe, and East Asia, with the total number of treated patients exceeding 450,000 (8). Emerging markets, particularly China, have become the main drivers of new proton center projects globally. Today, proton therapy is not only an indicator of a country’s advanced radiotherapy capabilities, but its clinical applications have also expanded from traditional pediatric tumors and skull base tumors to common solid tumors such as lung cancer, liver cancer, and prostate cancer.

Against the backdrop of rapid technological iteration and growing clinical demand for proton therapy, this article will systematically review recent key technological advances in the field, focusing on compact superconducting accelerators, PBS, AI-powered IGPT and motion management, as well as FLASH proton therapy. Additionally, this article will summarize the global development status of proton centers and provide an in-depth discussion of the localized practices, cost accessibility, and future sustainable development pathways of proton therapy in China. The aim is to offer a reference for promoting the development of proton therapy systems toward a more accessible, intelligent, and high-value precision radiotherapy framework.

## 2 Breakthroughs in core proton therapy technologies

The rapid expansion of the clinical application scope of proton therapy in recent years has been fundamentally driven by a series of breakthrough technological advancements. These innovations, centered on the three core directions of miniaturization, intelligence, and precision, have systematically addressed the technical bottlenecks in key areas such as equipment integration, beam delivery, accuracy verification, and treatment planning in traditional proton therapy, collectively establishing the foundation of the modern proton therapy technology system.

### 2.1 Accelerators and system integration

The core component of a proton therapy system is the accelerator, and its technological progress directly determines the system’s spatial layout, construction and operational costs, beam quality, and ability to meet complex treatment needs. It is one of the key factors influencing the accessibility and clinical adoption of proton therapy. In clinical proton therapy accelerators, proton ion sources (such as duoplasmatron, microwave, or radiofrequency (RF) ion sources) produce protons (H^+^) by ionizing hydrogen gas, which are then injected into the accelerator for further acceleration (9). The efficiency and stability of the ion source are critical for achieving the beam current and quality required for clinical applications. From a developmental perspective, the evolution of proton therapy largely represents a history of innovation in accelerator miniaturization, efficiency, and intelligence. The main types of proton accelerators currently used in clinical practice include synchrotrons and cyclotrons, each with distinct advantages and limitations. Synchrotrons enable active adjustment of proton beam energy (e.g., 70–250 MeV) by varying the magnetic field strength. This on-demand energy supply method produces almost no additional neutrons and facilitates expansion into multi-particle treatment modes such as carbon and oxygen ions. However, the system structure is more complex, and the beam output is pulsed (9, 10). Cyclotrons, on the other hand, produce a continuous proton beam at a fixed energy (e.g., 230–250 MeV), which is then degraded via an Energy Selection System (ESS) to meet the treatment requirements of tumors at different depths. Although cyclotrons offer high beam intensity and stable operation, the energy degradation process generates secondary neutron radiation, affecting the surrounding environment and equipment maintenance (11, 12). It is important to emphasize that both types of accelerators can achieve extremely high treatment precision, and the impact of beam quality differences on the final clinical outcome is far less significant than other factors such as patient positioning and motion management (13). Nevertheless, the large footprint and high construction costs associated with traditional accelerators remain major bottlenecks hindering the widespread adoption of proton therapy.

To overcome this challenge, compact/single-room proton therapy systems have emerged as a significant breakthrough in proton therapy equipment in recent years (14). These systems have revolutionized space requirements through the integration of superconducting technology and optimized structural design. Representative examples include the IBA Proteus ONE and Varian ProBeam 360°systems. While they retain the traditional accelerator-beamline-gantry architecture, the adoption of more compact superconducting cyclotrons, shorter beamlines, and highly integrated control systems successfully consolidate the core functionalities of multi-room systems into a single-room configuration (15, 16). The Mevion S250i system employs the world’s first superconducting synchrocyclotron, innovatively integrating the accelerator directly onto the rotating gantry, achieving an integrated “accelerator-gantry” design that allows the entire device to fit within a standard radiotherapy room (17). The IBA Proteus^®^ONE system also employs a superconducting synchrocyclotron, significantly reducing the system footprint and construction costs (18). The domestically developed compact superconducting cyclotron proton accelerator, developed by the Chinese Academy of Sciences Hefei Institutes of Physical Science, with a diameter of only 2.2 meters and a total weight not exceeding 50 tons, is one of the most compact proton accelerators globally (19). The widespread application of these compact systems has reduced the construction cycle of proton centers from 3–5 years to 1–2 years, with significantly lower overall costs, greatly promoting the global popularization and regional deployment of proton therapy.

Looking ahead, with the rapid development of new material technologies such as high-field superconducting magnets and novel acceleration mechanisms like laser-plasma acceleration, proton accelerators are evolving towards lighter, more intelligent, and lower energy consumption designs (20). Sumitomo Heavy Industries in Japan has developed a specially designed cyclotron capable of simultaneously outputting the high-energy, low-current beam required for proton therapy and the low-energy, high-current beam needed for Boron Neutron Capture Therapy (BNCT), enabling the sharing of two advanced radiotherapy techniques on a single accelerator platform (21). Concurrently, proton-heavy ion synchrotrons, serving as integrated treatment platforms supporting “dual-treatment with one machine,” are being rapidly developed and clinically deployed worldwide. Japan, Germany, and China have taken the lead in this area, completing prototype validation and clinical platform construction, representing the future trend of multi-species platformization and the integration of treatment and research in particle therapy systems (22). Notably, due to their continuous high-intensity beam characteristics, cyclotrons are considered an ideal platform for achieving FLASH radiotherapy (23). Meanwhile, laser-plasma accelerators can generate ultra-short proton pulses providing instantaneous dose rates up to 10^^7^ times higher than traditional continuous proton beams, offering a unique technical means for investigating the biological effects of FLASH radiotherapy (24). In the future, proton accelerators are expected to achieve further miniaturization, even desktop-sized systems, and may evolve into integrated platforms combining multi-modal radiotherapy technologies, opening up broader possibilities for scientific research and clinical applications. **Table 1** summarizes a comprehensive comparison between synchrotrons and cyclotrons.

**Table 1:** Synchrotron vs. cyclotron vs. synchrocyclotron accelerator comparison.

Feature	Synchrotron	Cyclotron	Synchrocyclotron
Beam Type	Pulsed Beam	Continuous Beam	Pulsed Beam
Energy Variation	Internal adjustment, directly accelerated to the desired energy	External energy degradation, energy selection via ESS	Variable energy, achieved by adjusting the RF frequency.
Energy Switching Time	Relatively slow (~100 ms – 1 s)	Very fast (~10–100 ms)	Fast (~10–100 ms)
Advantages	High energy efficiency, superior beam quality, lower secondary neutron radiation	High average beam intensity, high treatment throughput, stable system operation	Compact size, facilitating integration and maintenance.
Construction/Operational Cost	High cost, complex system, high maintenance requirements	Lower/Moderate cost, short construction time for compact systems	Low cost, single-room configuration
Emerging Technology Potential	Proton-heavy ion (e.g., carbon, oxygen) integration	Proton-BNCT integration, FLASH radiotherapy, ultra-compact design	Single-room configuration, desktop-sized, and integrated with the rotating gantry
Current Status & Trend	Preferred choice for large academic/research centers	Market leader, especially in the compact/single-room system market	The mainstream technology of single-room compact systems
Representative Vendors & Models	Hitachi: PROBEAT Mitsubishi: VERO	IBA: Proteus ONE Varian: ProBeam 360°	IBA: ProteusONE Mevion:S250i

### 2.2 Revolution in beam delivery technology

In the evolution of proton therapy, the Beam Delivery System (BDS) serves not only as the “last mile” connecting the accelerator to the patient but also as the core hub determining treatment precision, safety, and efficiency. Early proton therapy predominantly employed passive scattering techniques, relying on physical components such as scatterers, multi-leaf collimators, and compensators to shape the beam for target dose coverage (25, 26). Although mature and reliable, passive scattering—which successfully initiated the proton therapy era—exhibits inherent limitations: ①limited dose conformity and inability to perform intensity modulation, resulting in inadequate protection of proximal normal tissues for complex-shaped or concave targets; ②generation of substantial secondary neutrons due to interactions between scatterers/collimators and the proton beam, increasing neutron exposure to the patient and environment; ③dependence on patient-specific hardware, increasing preparation time and cost, thereby restricting clinical workflow efficiency; ④low beam utilization efficiency, as a large proportion of protons are blocked by collimators, leading to waste.

**Figure 1:**
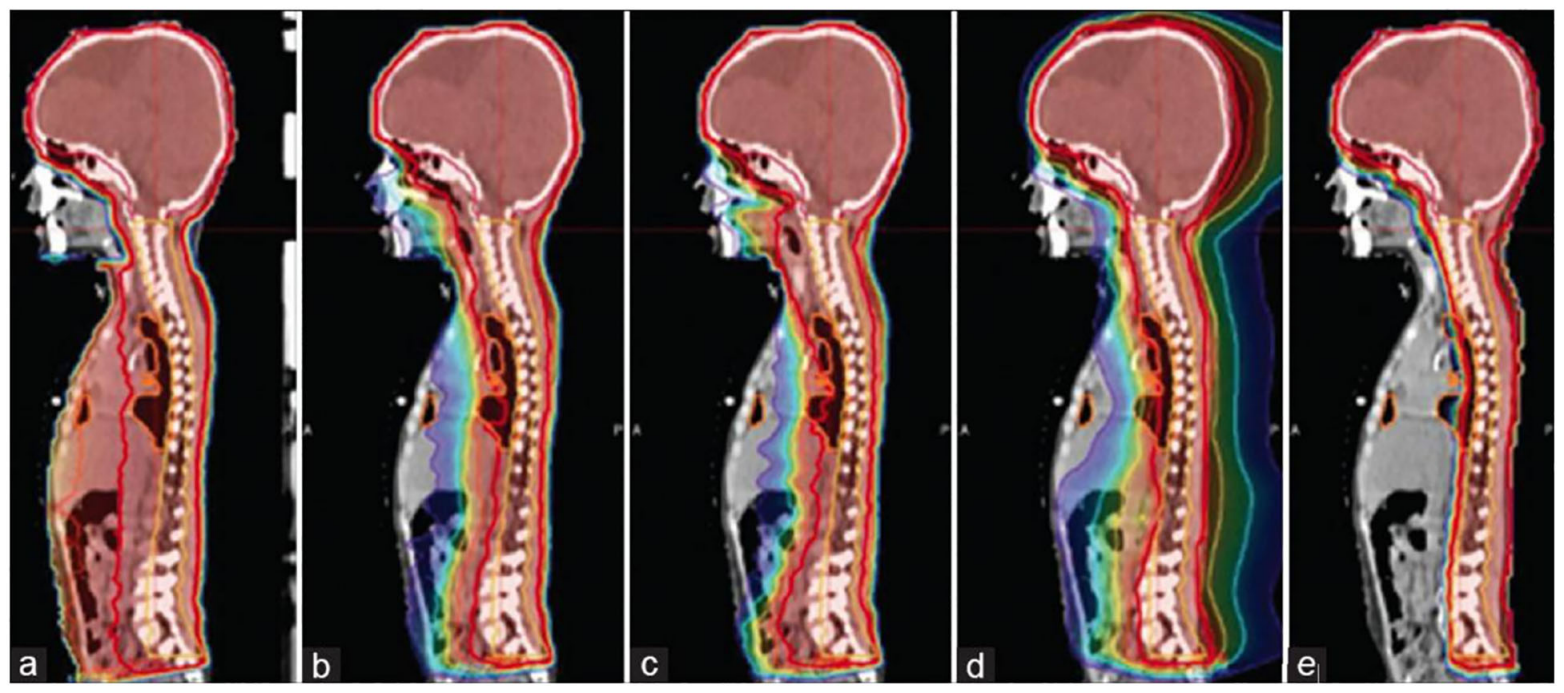
Dose distribution for a single pediatric patient. **(a)** Three-dimensional conformal radiotherapy; **(b)** IMRT; **(c)** VMRT; **(d)** Helical Tomotherapy; **(e)** PBS[34].

**Figure 2:**
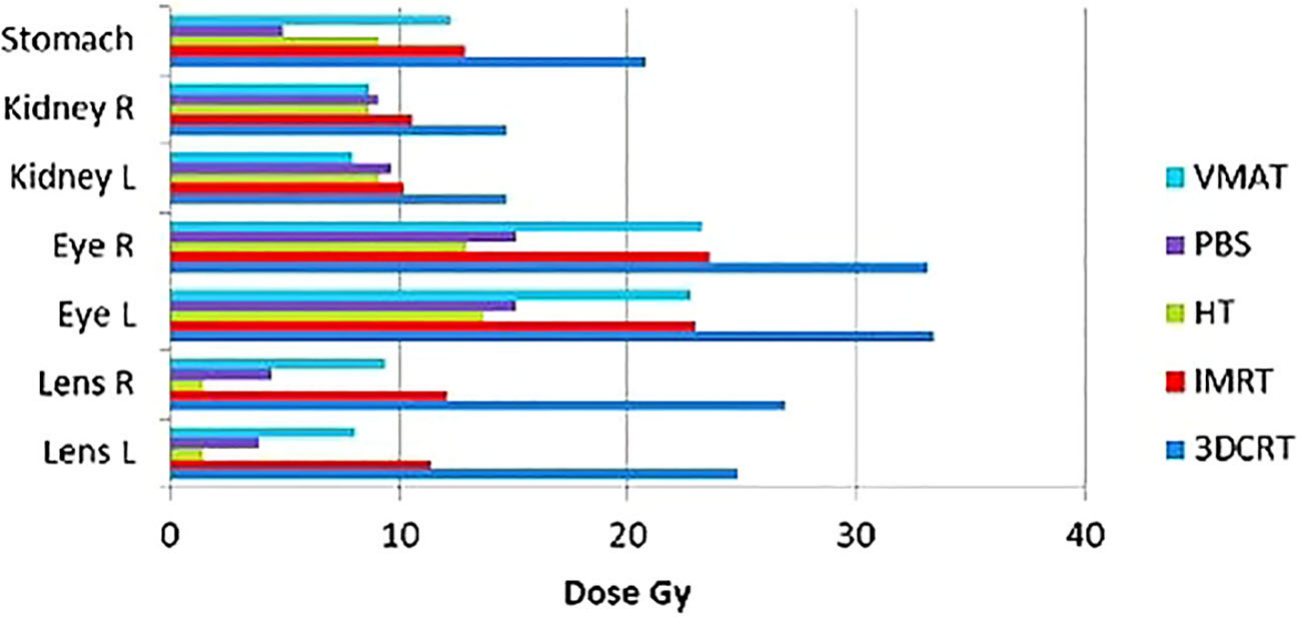
Equivalent uniform dose calculated for organ at risk for three-dimensional conformal radiotherapy, intensity-modulated radiotherapy, volumetric-modulated arc therapy, HT, and pencil beam scanning (34).

To overcome the limitations of passive scattering, Pencil Beam Scanning (PBS) emerged and has become the unequivocal mainstream of modern proton therapy. PBS completely eliminates the need for physical beam-shaping devices, employing instead high-speed magnetic deflection systems to actively and rapidly steer a pencil-sized proton beam, thereby creating a three-dimensional intensity-modulated dose distribution through “point-by-point, layer-by-layer, volume-based” delivery. This forms the foundational platform for Intensity-Modulated Proton Therapy (IMPT) (27, 28). PBS enables flexible and personalized control over the treatment target, with key advantages including: ①elimination of physical apertures, enhancing automation and adaptability to individualized therapies; ②support for high-precision directional scanning of complex target volumes (e.g., brainstem, hepatic portal, pelvic regions); ③significant reduction in treatment accessories (e.g., compensators, collimators), improving delivery efficiency and patient comfort; ④compatibility with Energy Layer Scanning (ELS) to achieve 3D dose shaping. Clinical studies have confirmed that for tumors adjacent to complex OARs, such as head and neck cancers and gliomas, IMPT offers significant advantages over photon-based IMRT/VMAT in reducing toxicities (29–31). Recent studies indicate that proton therapy also provides significant dosimetric advantages over photon-based techniques (IMRT/VMAT) in breast cancer treatment, particularly for left-sided cases and patients requiring regional nodal irradiation. Research by Prasanna et al. indicates that compared to IMRT (5.6 Gy), PT reduces the average mean heart dose (2.6 Gy) (32). Although proton therapy may be associated with a slightly higher incidence of mild skin toxicity, the rate of severe toxicities remains low with overall favorable cosmetic outcomes (33). The dosimetric advantages of proton therapy are expected to translate into reduced risks of cardiac and pulmonary toxicities, which is particularly crucial for long-term survivors. Furthermore, in pediatric craniospinal irradiation (CSI), IMPT demonstrates unparalleled dosimetric and clinical benefits. A study by Balasubramanian et al. showed that in Pediatric Craniospinal Irradiation, the mean esophageal dose was 4.73 Gy in the IMPT group, significantly lower than the 9.06 Gy in the IMRT group (34). Gram et al. reported that the mean dose for hippocampal avoidance was 14.7 Gy with IMPT, compared to 17.2 Gy with VMAT (35). This indicates the superiority of IMPT over IMRT/VMAT in protecting key brain regions such as the hypothalamic-pituitary axis and hippocampus, thereby helping to reduce cognitive and endocrine functional impairment (36). Multiple dosimetric studies have confirmed that IMPT in CSI significantly reduces the mean and maximum doses to critical organs such as the heart, lungs, liver, and kidneys, while also decreasing whole-body low-dose radiation exposure (37, 38). **Figure 1** shows the dose distribution for the single pediatric patient, and **Figure 2** shows the equivalent uniform dose for the organs at risk. Currently, nearly all modern proton systems, including the IBA Proteus, Varian ProBeam, and Mevion S250i, support or are configured by default with PBS.

Following the spatial precision achieved by PBS, a new and revolutionary dimension—time—has become the focus of reshaping the future of radiotherapy. This is embodied in Ultra-High Dose Rate (UHDR) radiotherapy, centered on the FLASH effect, which typically delivers a single fraction in an extremely short duration (< 200 ms) at an ultra-high mean dose rate (≥40 Gy/s, with many current studies using ≥100 Gy/s) (39). Numerous preclinical studies have remarkably demonstrated that, compared to conventional dose-rate irradiation at the same total dose, this extremely time-compressed delivery mode maintains equivalent tumor-killing efficacy while significantly reducing damage to surrounding normal tissues—a phenomenon termed the “FLASH effect” (40, 41). Current technological pathways to achieve FLASH-dose-rate proton delivery primarily include the transmission beam method and conformal FLASH (42). Among these, conformal FLASH combines the FLASH effect with the dosimetric advantages of proton therapy and represents the future direction (43). However, its technical challenges are immense, placing extreme demands on accelerator beam intensity, energy switching systems, scanning speed, and real-time dose monitoring and control (44). FLASH proton therapy remains in the preclinical research and early clinical trial stages. Several leading global proton centers (e.g., Cincinnati Children’s/UC Health in the USA, PSI in Switzerland) have successfully validated the FLASH effect in animal models and initiated the first clinical trials for human superficial tumors (e.g., skin cancer, extremity sarcomas) (45–47).

In modern proton radiotherapy systems, the beam is no longer merely a stream of particles but a multi-level systems engineering undertaking integrating physical control, shape design, biological dose optimization, and AI-driven process management. In recent years, across key dimensions such as precision control, layered scanning, dose modulation, and real-time feedback, proton beam delivery technology has undergone a major transformation—from scattering to scanning methods, from 2D irradiation to 4D adaptation, and from physical dose control to biological dose regulation—propelling proton therapy into an era of precision, intelligent control, and adaptability. **Table 2** summarizes a comparison between traditional and FLASH beam delivery modalities.

**Table 2:** Conventional vs. FLASH beam delivery comparison.

Feature	Conventional beam delivery	FLASH beam delivery
Core Objective	Improved Dose Conformity, Delivery Precision	Ultra-High Dose Rate, FLASH Effect
Dose Rate	1–10 Gy/min (~0.1 Gy/s)	≥40 Gy/s (typically 100–1000 Gy/s)
Irradiation Time	Tens of seconds to minutes	Sub-second (<1 s)
Technical Approach	Passive Scattering, Pencil Beam Scanning	Transmission Irradiation, Ultra-Fast Scanning
Key Challenges	Motion Management, Dose Accuracy, Conformity	Beam Current Control, UHDR Dosimetry, Ultra-Fast Scanning
Dosimetric Characteristics	Bragg Peak (Excellent)	Transmission (Poor), Conformal (Excellent)
System Complexity	High	Extremely High (requiring breakthroughs in multiple technical bottlenecks)
Clinical Stage	Routine Clinical Use Worldwide	Early-Stage Clinical Exploration/Trials

### 2.3 Multimodal image fusion and ai-enhanced IGPT technology

The precision of proton therapy depends not only on advanced accelerators and beam delivery systems but also critically on real-time accurate localization of the tumor target and dynamic motion management during treatment (48). Particularly in IMPT, where the treatment plan relies heavily on the precise coordination of particle beam depth and spatial positioning, even sub-centimeter tissue displacement can cause significant deviation of the delivered dose distribution from the original plan, compromising target coverage and increasing dose to normal organs. Current proton therapy primarily relies on Cone-Beam CT (CBCT) for patient setup verification and target localization. This involves using a mounted kV X-ray source and a flat-panel detector rotating around the patient to acquire data and reconstruct a 3D volumetric image at the treatment position (49). Currently, CBCT is a standard feature on most newly installed proton therapy systems. Some advanced systems (e.g., ProBeam 360, IBA Proteus ONE) integrate CBCT with digital X-ray dual-mode image guidance and support automated couch correction after registration, enhancing positioning efficiency (50). However, these techniques have limitations, including low soft-tissue contrast, additional radiation dose, and the inability to monitor intrafraction motion in real-time.

To overcome these bottlenecks, the next generation of MRI-Guided Proton Therapy (MRgPT) technology is rapidly developing towards multimodal fusion, functionalization, and real-time capability. MRI offers the advantages of no ionizing radiation dose, excellent soft-tissue contrast, and real-time dynamic imaging capability, allowing clear visualization of tumor and surrounding normal tissue morphology and motion. Integrating MRI with proton therapy systems presents significant technical challenges, primarily related to the mutual interference between strong magnetic fields and beam control, and the integration of the beamline nozzle with the MRI scanner (51, 52). The German OncoRay center successfully integrated a 0.22 T open low-field MRI system with a PBS system and demonstrated that good-quality MRI images could be acquired during proton beam irradiation, establishing the proof-of-principle for real-time MRgPT (53). Furthermore, Fujii et al. in Japan have proposed a conceptual design for integrating PBS into a 0.3T superconducting open MRI system, contributing to the initial design phase for MRgPT (54). MRI guidance is particularly suitable for tumor sites with significant motion (e.g., abdominal, pelvic tumors), enabling adaptation to anatomical changes during the treatment course through daily or even intrafraction adaptive replanning (55). To overcome the limitations of conventional fixed imaging systems (such as stationary CBCT) while avoiding the magnetic field integration challenges of MRI-guided therapy (MRgPT), mobile CT scanners are emerging as a crucial technological direction for enhancing the flexibility of online image-guided proton therapy (IGPT) (56). Research indicates that mobile CT provides superior soft tissue contrast and geometric accuracy compared to traditional orthogonal X-ray or cone-beam CT, which is particularly important for sites with significant anatomical variations such as the head and neck, pelvis, and thorax (57). This mobility and rapid imaging capability, especially for existing proton centers unable to undertake expensive large-scale gantry/beamline retrofits, offers a highly cost-effective pathway for online adaptive upgrades. Studies have demonstrated that its image quality is sufficient to support AI-driven rapid image registration and dose verification, effectively addressing Bragg peak drift caused by inter- and intra-fraction anatomical variations (58). As a result, the threshold for deploying online adaptive radiotherapy systems is significantly lowered. By integrating images acquired from mobile CT with the treatment planning system and performing rapid registration and dose verification assisted by AI algorithms, mobile CT effectively advances proton therapy toward more adaptive and accessible real-time guidance. Furthermore, a new generation of PET-guided proton therapy (PETgPT) is rapidly developing, becoming a crucial direction for the precision and adaptive evolution of proton therapy. Proton beams interacting with atomic nuclei in tissue generate short-lived positron-emitting nuclides (such as ^15^O, ^11^C, ^13^N). PET imaging can indirectly reflect the actual stopping position and range of protons, enabling comparison between planned and actual dose distributions, thus achieving online range verification and adaptive adjustments (59, 60). For instance, a hybrid imaging system developed through a European multi-center collaboration can already acquire positron signals in real-time during treatment, achieving sub-minute range monitoring (61). Recent research has also explored the deep integration of AI and PET imaging, using deep learning algorithms to denoise and reconstruct PET signals and predict dose distributions, significantly enhancing the accuracy and efficiency of PET-guided proton therapy (62). In the future, PETgPT is expected to collaborate with multimodal imaging systems like MRgPT and CBCT, enabling comprehensive, holographic adaptive management throughout the entire proton therapy workflow, thereby advancing proton therapy towards higher levels of precision and personalization.

AI, particularly deep learning, is addressing numerous traditional bottlenecks in multimodal image processing, significantly enhancing the efficiency and accuracy of image guidance. Deep learning-based auto-segmentation models (e.g., U-Net, RefineNet) can learn from large datasets annotated by radiation oncology experts to achieve fast, accurate, and highly consistent automatic contouring of tumor targets and Organs at Risk (OARs) on CT, MRI, and other images (63–67). This not only liberates physicians from time-consuming manual contouring and reduces inter-observer and inter-patient variability but also lays the groundwork for fast online adaptive radiotherapy (OART). Rigid registration (global alignment) and deformable registration (local alignment) between multimodal images are crucial yet challenging aspects of fusion (68). AI algorithms can learn from large sets of aligned image pairs to accurately predict complex deformation fields, achieving high registration accuracy even when appearance differs greatly across imaging modalities (69). Tomar et al. achieved excellent results in cross-modality segmentation between MRI and CT data in an unpaired multimodal brain tumor dataset using an adversarially trained image translation model (70). AI-driven multimodal image registration algorithms can accurately align CT, MRI, and PET images, enabling the fusion of functional and anatomical information. AI can also effectively suppress artifacts in CBCT images, enhancing online image quality. For instance, metal implants or patient motion during treatment can cause artifacts in CBCT images, degrading image quality and registration accuracy. AI algorithms based on Generative Adversarial Networks (GANs) or diffusion models can effectively suppress these artifacts, improving the signal-to-noise ratio and clarity of low-quality online images, thereby providing a more reliable basis for precise guidance (71, 72). Furthermore, AI can extract deep quantitative radiomic features from multimodal images and integrate them with clinical and genomic data to build predictive models (73, 74). Examples include predicting the Relative Biological Effectiveness (RBE) of protons in tumors, radiosensitivity, or the risk of post-treatment recurrence, thereby advancing proton therapy from physical precision towards biological precision (75).

Currently, the deep integration of multimodal image fusion and AI is reshaping the future landscape of image-guided proton therapy: ①Fully intelligent OART: AI will integrate daily CBCT/MRI images, automatically perform image segmentation, dose recalculation, plan optimization, and verification/comparison, completing the entire adaptive process within minutes, making “see-and-treat” a routine practice (76); ②Multimodality-guided FLASH therapy: Guidance systems combining ultra-high temporal resolution imaging with AI predictive models hold the potential to determine the target and manage motion within sub-second timescales, ensuring FLASH therapy is both fast and accurate (77); ③Cross-institutional knowledge sharing and Federated Learning: To address the problem of medical data silos, federated learning allows multiple proton centers to collaboratively train AI models without sharing raw patient data. This will greatly facilitate the development of robust, generalizable, and privacy-preserving AI-guided models, promoting the dissemination of best clinical practices (78). In summary, the two technological engines of multimodal image fusion and AI enhancement are profoundly empowering IGPT, driving its evolution from geometric guidance towards biological guidance and intelligent operation. They not only address key bottlenecks in current clinical practice but also lay a solid foundation for tackling future complex clinical challenges and innovative therapies (e.g., multi-particle therapy, FLASH radiotherapy).

### 2.4 Intelligent motion management and AI-enhanced technology

With proton therapy entering the era of three-dimensional precision control and IMPT, the motion of tumors and OARs during treatment has increasingly become a core challenge limiting the precision, safety, and consistency of proton therapy outcomes. Particularly in thoracic and abdominal cancers (such as lung cancer, liver cancer, pancreatic cancer, etc.), dynamic factors such as respiratory motion, gastrointestinal peristalsis, and bladder filling can cause centimeter-level changes in target position and shape. This can easily lead to misplacement of the Bragg peak, deviation from the planned dose distribution, and even cause dose hot/cold spots and excessive irradiation of OARs. To address these uncertainties, conventional proton therapy typically employs:①Positioning/Respiratory Motion Management Techniques: These include Deep Inspiration Breath Hold (DIBH) and Active Breathing Control (ABC). ABC is a closed-loop technology that stabilizes diaphragm position by precisely controlling the patient’s inspiratory depth, widely used in proton therapy to manage target displacement in abdominal organs such as the liver and pancreas (79). DIBH increases the distance between the lungs and heart by requiring the patient to hold their breath at maximum inspiration (80). ②Surface-Guided Technology: Surface Guided Radiation Therapy (SGRT), primarily utilizing infrared surface tracking systems, is extensively applied in proton therapy. SGRT enables real-time, non-contact monitoring of patient surface motion, thereby accurately verifying if the patient maintains a preset DIBH position or managing small-range motion drifts in real-time tracking mode (81). Although these conventional methods are beneficial for ensuring target coverage, they still face limitations such as restricted patient applicability, high demands on patient compliance, and system response delays. This not only diminishes the core advantage of proton dose conformity but also fails to fully meet the demands of complex targets and high-dose modulation. Consequently, developing a holistic, intelligent, closed-loop motion management system has become a key focus for advancing precision in current proton therapy. In recent years, proton therapy motion management technology has achieved several breakthrough advancements. Firstly, gating technology has become one of the routine clinical techniques. Gating can be combined with various monitoring methods such as surface tracking, X-ray fluoroscopy, or implanted fiducial markers to enhance motion adaptability and treatment precision (82). The SDX^®^ Respiratory Gating System has been widely adopted for respiratory motion management in proton therapy. It enables real-time monitoring and control of patient breathing, allowing gated delivery of the proton beam during specific phases of the respiratory cycle (48). This integrated approach has become an advanced practice in proton therapy for thoracic and abdominal tumors—such as lung cancer, liver cancer, and left-sided breast cancer—with the objectives of maximizing normal tissue sparing and enhancing treatment precision and efficacy (46, 83, 84). Secondly, novel motion mitigation techniques like layer repainting and volumetric repainting have been widely adopted in PBS. These techniques can significantly reduce interplay effects, improving dose homogeneity and plan robustness for moving targets (85, 86). Latest research demonstrates that combining gating with repainting techniques can reduce motion-induced dose inhomogeneity to below 5%, substantially enhancing the safety and efficacy of proton therapy for thoracic and abdominal tumors (87). Therefore, developing comprehensive, intelligent, closed-loop motion management systems has become a key focus of innovation for improving precision in proton therapy.

The introduction of AI technology is fundamentally changing the paradigm of motion management, shifting it from passive adaptation to active prediction and real-time response. By analyzing multimodal dynamic imaging data with deep learning algorithms, AI can construct individualized respiratory motion models and achieve sub-second prediction of target displacement (88, 89). For example, prediction models based on Long Short-Term Memory (LSTM) networks and Spatio-Temporal Convolutional Neural Networks (ST-CNN) can learn the nonlinear mapping relationship between patient surface optical monitoring signals and the position of internal fiducial markers, enabling real-time inference of the target’s 3D motion trajectory. Under specific conditions, the prediction accuracy can reach the millimeter level, with significantly reduced time latency compared to traditional methods (90, 91). The core breakthrough of the new generation of motion management systems lies in achieving closed-loop “imaging-AI-beam” control. Research by Dedes et al. emphasizes the critical role of Monte Carlo in proton therapy dose calculation. Models trained through federated learning frameworks can online update the target deformation field and trigger dose recalculation (92). For instance, when target displacement exceeding a preset threshold is detected, the system can automatically perform Monte Carlo dose calculation and verification for a new plan and adjust the PBS path accordingly, achieving true dynamic target tracking irradiation. The precision assurance system of proton therapy is undergoing a profound transformation. It is no longer merely static setup calibration but has evolved into an intelligent closed-loop system integrating 4D time resolution, inter-fraction dynamic adaptation, and AI-driven control. The integration of IGPT and motion management technology, by establishing an intelligent perception-prediction-response closed loop, not only effectively addresses the challenges posed by physiological motion but also provides key technical support for achieving personalized, real-time adaptive precision proton therapy. With the synergistic development of algorithmic innovation and hardware advancement, intelligent motion management will become a standard feature of the next generation of proton therapy systems, ultimately delivering a safer and more effective treatment experience for patients.

### 2.5 Treatment planning and quality assurance techniques

With the continuous advancement of proton therapy technology, treatment planning methods and quality assurance systems are also undergoing iterative upgrades, becoming core components for achieving high-precision, individualized, and safe treatment. Modern proton therapy planning not only pursues optimal dose distribution but also emphasizes the active management of uncertainties and precise coverage of complex indications. Current clinical proton therapy planning primarily includes three mainstream methods: Single Field Optimization (SFO), Multi-Field Optimization (MFO), and Robust Optimization. SFO (also known as Single Field Uniform Dose, SFUD) involves independent optimization of each irradiation field, making it suitable for cases with regular target shapes and minimal motion impact, offering strong robustness and simplicity (93). MFO forms the basis of Intensity-Modulated Proton Therapy (IMPT), achieving highly conformal dose distributions and OAR sparing through synergistic multi-field optimization. It is particularly suitable for complex targets and cases adjacent to critical organs but is more sensitive to setup and range uncertainties (94). Robust Optimization directly incorporates various uncertainty factors, such as patient positioning and range, during the planning stage. By ensuring target coverage and OAR protection under various error scenarios, it has become the mainstream method recommended by international guidelines, especially for demanding situations like head and neck, pediatric, and moving targets (95, 96).

In recent years, Proton Stereotactic Body Radiation Therapy (PSBRT) has developed rapidly. Leveraging its high dose per fraction, few fractions, and excellent OAR sparing capabilities, it has demonstrated excellent local control rates and low toxicity in various solid tumors such as lung cancer and liver cancer (97, 98). Proton Stereotactic Radiosurgery (Proton SRS) has been increasingly applied in recent years for indications such as intracranial benign and malignant tumors and brain metastases (99). Compared to photon-based SRS, Proton SRS achieves a sharper dose fall-off and superior OAR sparing, making it particularly suitable for pediatric cases and complex anatomical locations (100, 101). Spatially Fractionated Radiation Therapy (SFRT), such as proton lattice therapy, creates alternating high and low dose regions within the tumor, triggering immune responses and bystander effects, offering new strategies for refractory large-volume tumors (102, 103). Proton Microbeam Radiation Therapy (pMRT), as an emerging direction, utilizes high-dose proton microbeams with submillimeter to micrometer-scale widths to achieve spatial fractionation of high- and low-dose regions within the tumor. Preliminary animal studies and dosimetric research have demonstrated its unique advantages in normal tissue protection and eliciting immune responses (104, 105). Proton Arc Therapy (PAT) and 4π non-coplanar proton therapy are currently internationally leading directions for dosimetric innovation. PAT achieves multi-angle arc irradiation through continuous gantry rotation, significantly improving dose conformity and robustness while reducing OAR dose, making it particularly suitable for complex or moving targets (106). 4π proton therapy utilizes multiple non-coplanar beams to maximize dose concentration and OAR avoidance. Preliminary studies show its unique advantages in complex sites like the brain and head and neck (107). Additionally, upright proton therapy with a fixed gantry represents an innovative model for new-generation compact centers. By positioning patients upright and using fixed beam delivery, it significantly reduces equipment volume and construction costs, while also helping to reduce organ motion and improve treatment comfort and efficiency (108).

It is noteworthy that the biological effects of proton therapy are closely associated with linear energy transfer (LET) (109). In recent years, LET optimization and evaluation have become integral components of proton treatment planning (110, 111). Clinically, the treatment planning system can generate LET distribution maps to assess the spatial relationship between high-LET regions and critical structures such as OARs and the central nervous system (CNS). For central nervous system cases, if high-LET regions are proximate to critical functional areas, adjusting the beam arrangement is recommended to mitigate potential biological damage risks, thereby achieving dual optimization of both physical dose and biological effect (112).

High-precision proton therapy places higher demands on Quality Assurance (QA). Modern QA systems encompass the entire process, from equipment calibration and beam parameter verification to plan dosimetric verification and patient-specific QA. For PBS and IMPT, QA focuses include spot position and energy layer verification, dose distribution consistency, and end-to-end testing (113). In recent years, new methods such as secondary dose calculation based on Monte Carlo algorithms, treatment log file analysis, and 3D dose verification have become mainstream (114, 115). The introduction of AI-assisted QA tools can automatically identify discrepancies between planned and delivered doses, enhancing QA efficiency and safety (116). For high-dose, few-fraction SBPT and arc therapy, the QA system must also pay attention to dose accuracy in high-dose gradient regions and the real-time performance of motion management. New technologies like spatial fractionation and 4π therapy also pose multi-dimensional and dynamic challenges for QA, driving the development of adaptive QA systems based on big data and intelligent algorithms. In summary, the continuous innovation in treatment planning and quality assurance not only provides a solid guarantee for the high precision and safety of proton therapy but also lays the foundation for diverse, complex clinical needs and frontier technology exploration. In the future, with the deep integration of AI, automation, and multimodal imaging, proton therapy planning and QA systems will become more intelligent, efficient, and individualized, propelling precision radiotherapy to new heights.

## 3 Global clinical application status

The ultimate goal of technological breakthroughs in proton therapy is to serve clinical practice and improve patient outcomes. Leveraging its unique physical advantages, proton therapy has steadily evolved from an initial physics marvel into an indispensable clinical tool in modern radiotherapy. This chapter will systematically review its global development trends and focus on characteristics in China to present a comprehensive picture of its clinical applications.

### 3.1 Global distribution and development status of proton centers

Driven by both technological maturity and clinical demand, the construction of global proton therapy centers has entered a period of rapid development. According to the latest statistics from PTCOG, by the end of 2024, there were over 120 operational proton therapy centers worldwide, with more than 50 additional centers under construction or in the planning stages. The cumulative number of treated patients has exceeded 450,000 (8). Of these, China now has more than 30 proton centers operational or under construction, accounting for over half of all new global projects and becoming the primary driver behind the worldwide expansion of proton centers. This growth trend clearly demonstrates the transition of proton therapy from the ivory tower of a few top physics laboratories to a wave of popularization in mainstream cancer centers globally. Global proton centers exhibit a distinct tripartite distribution pattern, concentrated mainly in three major regions: North America, Europe, and East Asia, each with distinct regional characteristics:

①North America: The United States has the largest number of proton therapy centers and the richest clinical experience globally. Many of its leading centers, often affiliated with world-leading cancer research institutions (e.g., MD Anderson Cancer Center) and large healthcare groups, forming integrated hubs for research, clinical practice, and education. These centers not only handle high patient volumes but also lead globally in exploring new indications, standardizing techniques, and developing combination therapies (e.g., proton therapy combined with immunotherapy) (117, 118). ②Europe: Particle therapy in Europe has a long history and a relatively balanced distribution. Countries like Germany and France host multiple world-renowned proton centers. Europe excels particularly in multi-particle platforms (e.g., the integrated proton/heavy-ion technology at Heidelberg Ion Beam Therapy Centre, HIT, Germany) and the development of new technologies (e.g., spot-scanning technology at the Paul Scherrer Institute, Switzerland), making its centers not only treatment facilities but also important hubs for technical R&D and physics research (23, 119). ③Asia: Led by Japan and China, East Asia is the region with the fastest growth in proton therapy in recent years (120–122). Japan started early, possesses deep technical expertise, rich clinical experience, and mature application of compact equipment. Furthermore, its relatively lower treatment costs compared to Europe and America attract international patients. China has become the primary engine for the recent growth in proton centers, with national and provincial-level large-scale proton centers being established successively. The Shanghai Proton and Heavy Ion Center, as China’s first such facility, has consistently treated over 1000 patients per year with a single device for four consecutive years, being the first proton/heavy-ion center globally to achieve this milestone, demonstrating both “China speed” and operational efficiency (123). Given its vast patient base, China is expected to become the largest proton therapy market worldwide within the next few years. To more intuitively display the global landscape, **Table 3** summarizes the development status of proton centers in major regions and countries.

**Table 3:** Status of global proton/particle therapy centers by country/region (as of the End of 2024).

Region	Country/territory	Operational (Approx.)	Under construction/planned (Approx.)	Regional characteristics & trends
North America	USA	45+	10+	World’s largest market, technology innovation leader. Mature network from academic centers to community single-room systems.
	Canada	1	1	Relatively cautious development, but flagship centers are operational.
Europe	Germany	8	2	Technologically advanced, hosts multiple integrated proton/heavy ion centers.
	UK	4	1	Led by the National Health Service (NHS), features large public centers supplemented by private ones.
	France	4	2	Steady growth, emphasizes clinical research and multi-center collaboration.
	Switzerland	2	1	Home to the renowned Paul Scherrer Institute (PSI), a pioneer in scanning technologies.
	Other European Nations	15+	10+	Italy, Netherlands, Spain, Austria, among others, have established centers.
East Asia	China	10+	20+	Fastest-growing emerging market globally, rapid construction pace. Domestic equipment breakthroughs, immense future market potential.
	Japan	25+	5+	Highest per capita availability, leading technology. Strong expertise in both proton and heavy ions, widespread use of compact systems.
	South Korea	2	2	Early adopter, hosts Asia’s first proton center, high technical proficiency.
	Taiwan (China)	2	1	Hosts renowned centers like Chang Gung Memorial Hospital, rich clinical experience.
Other Regions	Australia	1	1	First proton center in the Southern Hemisphere operational.
	India	1	1	Major developing country entering the field, significant market potential.
	Middle East	1	2	Leveraging strong capital investment to build high-specification particle therapy centers.
Total	Global	~125	~55	

The construction pace of global proton therapy centers has accelerated significantly since 2010. The emergence of compact, single-room centers has notably reduced construction costs and space requirements, enabling proton therapy to decentralize from national-level large medical centers to regional hospitals, thereby greatly promoting technological popularization and regional distribution. In summary, the global distribution of proton therapy centers is shifting from high concentration to multi-point diffusion, with the growth momentum transitioning from being technology-driven to being dually driven by clinical demand and accessibility. Looking ahead, as the market potential in populous countries such as China and India is further tapped, and with continued technological miniaturization and cost reduction, the global growth curve of proton centers is expected to maintain a steep upward trajectory.

### 3.2 Characteristics and development status of clinical applications in China

As the fastest-growing emerging force in the global proton therapy field, China’s clinical applications demonstrate distinct late-mover advantages and Chinese characteristics. Progressing from initially importing foreign systems, to gradually achieving domestic production of accelerators, beam control systems, and treatment planning systems, and further forming a development pattern that balances research-oriented and application-oriented centers under public leadership and industrial collaboration, China’s proton therapy is accumulating a technology system and clinical application features with local characteristics. Within just over a decade, China has not only completed the leap from scratch to large-scale hardware construction but has also accumulated valuable local experience in clinical practice, technology R&D, and indication exploration, tailored to its national conditions and high-incidence tumor profiles. It is gradually transitioning from a technology follower to a leader in certain fields. Unlike the gradual development path of European and American countries, China’s proton center construction is characterized by high starting points, rapid speed, and large scale. Since the official operation of the first proton-heavy ion center in mainland China (Shanghai Proton and Heavy Ion Center) in 2015, the construction of proton centers nationwide has entered the fast lane. Driven by both national policy support and huge market demand, regions like Beijing, Shanghai, Guangdong, and Shandong have made significant investments, with the number of centers under construction or planning ranking among the top globally (124). Benefiting from the late-mover advantage, nearly all newly built proton centers in China have skipped early technologies like passive scattering, directly equipping the most advanced PBS technology and integrated image-guided systems. Some centers have even proactively planned proton-heavy ion integrated platforms (e.g., the Guangzhou Shunde Heyou Hospital successfully achieved accelerator beam extraction in July 2025), ensuring their technical level aligns directly with top international standards (125). In recent years, national teams represented by the Hefei Institutes of Physical Science, Chinese Academy of Sciences, and China General Nuclear Power Group, as well as commercial companies like United Imaging Healthcare and Mevion Medical Systems, have made major breakthroughs in the independent R&D of core components such as compact superconducting cyclotrons, rotating gantries, and treatment planning systems (126). The advent of domestically produced proton therapy systems has not only broken the technological monopoly of foreign manufacturers but is also expected to significantly reduce equipment costs and operational expenses, laying a solid foundation for the further popularization and decentralization of proton therapy in China (127).

China’s clinical practice closely focuses on its domestic high-incidence tumor spectrum, accumulating world-leading clinical experience in treating characteristic cancers such as nasopharyngeal carcinoma, glioma, and esophageal cancer, and contributing high-quality Chinese evidence to international particle therapy (128–130). The ten-year clinical outcomes of domestic centers, represented by the Shanghai Proton and Heavy Ion Center, are particularly outstanding. Their published clinical data show a 5-year overall survival rate of 92.9% for newly diagnosed nasopharyngeal carcinoma patients, demonstrating significant advantages over traditional IMRT in reducing severe complications such as radioactive brain injury, xerostomia, hearing impairment, and trismus; a 5-year overall survival rate of 70.3% for early-stage (Stage I) non-small cell lung cancer patients, and a 5-year cause-specific survival rate of 100% for localized prostate cancer patients; for pancreatic cancer, often regarded as the “king of cancers,” the 3-year overall survival rate reached 29.4% (131). The incidence and severity of toxic side effects were significantly reduced in all treated patients. The clinical data in this field have become an important contribution from China to the global proton therapy community. With socioeconomic development and increased health awareness, the standardized treatment of pediatric tumors is also receiving growing attention. The recognized advantages of proton therapy for pediatric tumors make it a key development direction for major new proton centers in China. Proton centers in Beijing, Shanghai, and other locations have established close collaborations with top domestic children’s hospitals, systematically conducting proton therapy for pediatric solid tumors like neuroblastoma, medulloblastoma, and rhabdomyosarcoma, and actively participating in international multi-center collaborations (132). For other high-incidence tumors in China, such as lung cancer, esophageal cancer, and liver cancer, domestic centers are also actively exploring the clinical value of proton therapy and have initiated multiple prospective clinical studies, aiming to accumulate more high-level local evidence-based medical evidence for these indications. (133) In the future, China is not only poised to become the world’s largest proton therapy application market but also, leveraging its vast patient cohorts, rich experience with characteristic diseases, and cross-disciplinary advantages in AI and big data, has the potential to contribute more high-quality evidence, novel approaches, and unique insights to global clinical research and technological innovation in proton therapy.

## 4 Conclusion and challenges

Driven by key technological breakthroughs, proton therapy has completed a paradigm shift from a frontier physics concept to a modern clinical tool. The technology system centered on compact superconducting accelerators and PBS has not only enhanced treatment precision but also significantly promoted its global adoption. Worldwide, the number of proton centers has exceeded 120, primarily distributed across North America, Europe, and East Asia, with China emerging as the fastest-growing emerging market. The range of treatable diseases has expanded from pediatric and skull base tumors to include lung cancer, liver cancer, among others, with leading experience accumulated in treating characteristic high-incidence tumors in China such as nasopharyngeal carcinoma. Concurrently, cutting-edge explorations represented by FLASH radiotherapy, multi-particle platforms, and AI-driven adaptive therapy are continuously broadening its technological boundaries, heralding a future of more precise and personalized adaptive treatments.

However, on the path toward becoming an accessible standard treatment, proton therapy still faces three core challenges. Foremost among these are economic feasibility and accessibility, where high costs and limited insurance coverage remain the most significant barriers to widespread adoption. Secondly, despite encouraging results, the clinical-cost effectiveness advantages of proton therapy over advanced photon therapy for a broader range of cancers still require validation through larger international multicenter RCTs and high-quality real-world data. Finally, future development must focus on overcoming technical integration and biological challenges. While current precision in proton therapy is primarily physical, the key to determining its future potential lies in advancing from geometric precision to biological precision—specifically, achieving variable RBE-guided dose optimization and ultimately overcoming technical hurdles such as MRgPT and FLASH radiotherapy. In summary, only through the coordinated advancement of technological innovation, clinical research, and health economic evaluation can the immense potential of proton therapy be fully realized as a universally accessible value for safeguarding global patient health.
